# Prey size as a critical factor for bird bone taphonomy in Eagle Owl (*Bubo bubo*) pellets

**DOI:** 10.1038/s41598-019-55721-7

**Published:** 2019-12-16

**Authors:** Anna Rufà, Véronique Laroulandie

**Affiliations:** PACEA - UMR 5199 CNRS, Université de Bordeaux, Bâtiment B2. Allée Geoffroy Saint Hilaire CS 50023, 33615 Pessac, Cedex France

**Keywords:** Palaeontology, Archaeology

## Abstract

Each predator hunts and consumes its prey in a particular way. Consequently, the traces left by predators on bones might vary according to the manner in which the prey are processed. For this reason, prey size has been proposed as a key issue that affects the damage inflicted on bones. The Eagle Owl (*Bubo bubo*) is one of the main potential predators of small prey found in archaeological sites. However, detailed taphonomic research describing bone accumulations produced by this nocturnal raptor is still scarce. The aim of the present work is to describe a modern accumulation of pellets originated by the Eagle Owl from this perspective, with a specific focus on birds. Particular attention is paid to prey size to evaluate the real significance of this variable when assessing bone damage. The results confirm that bone alterations reflect how prey was ingested, as the bones show greater damage with increasing prey size. This finding emphasises the complexity of characterising archaeological accumulations, as the alterations will vary according to prey size. In addition, bone architecture—or other aspects that cannot be controlled—may hinder accurate diagnosis and should be taken into account.

## Introduction

Archaeological assemblages are the result of different events taking part at the same site. The agents implicated in the formation of these assemblages can also be diverse, since hominids, raptors and mammalian carnivores^1–11^ could have used the same spaces to perform their activities. This is a challenging context when attempting to understand the processes involved in the formation of an archaeological site, as separating the palimpsest of occupation is almost impossible during the excavation. This emphasises the importance of identifying predators that could intervene in the makeup of the recovered remains.

Small animal remains have not often been a focus of interest for archaeozoologists, because there was a general assumption that they were not processed and/or consumed by prehistoric populations. This is why they passed unnoticed from further analyses, even though they could provide important information regarding occupational dynamics of a site and past human behaviour. During the last decades, interest has been growing in identifying the causes taking part in small prey accumulations and in distinguishing the part made by humans. This is mostly a result of the emergence of evidence of Palaeolithic small prey exploitation^10,12–27^. Concurrently, many published studies have been attempts to characterise accumulations made by small mammalian carnivores and raptors whose diets consist largely of leporids or birds, which are the most common small prey found in Europe. Particular interest has been focused on rabbits, as they are widely distributed throughout the continent^12,26,28–34^. Some studies have also been published on accumulations of bird remains originated by non-human predators. Nevertheless, the referential framework is still limited, and the works already published have attempted to assess possible patterns of anatomical representation and fragmentation of bones^35–39^. However, they do not pay special attention to the identification of other taphonomic traits that could help to determine the alterations originated by each predator type. Only a limited number of studies have attempted to assess bird accumulations from a taphonomic point of view, but this approach was confirmed as the most successful way to diagnose possible accumulators^1,6,40–42^.

Despite the recognition of the scientific importance of taphonomic data, most of this research has provided only a general characterisation of avian accumulations. By contrast, little attention has been paid to such key issues as the differences that might result from the high diversity of prey consumed by the pursuers. Thus, many factors can be considered when trying to determine predator accumulations. Among these, prey size is one of the main variables that would result in differential prey treatment. This variable has previously been introduced by some scholars^35,43–45^ who have observed many differences in prey modifications depending on the prey size.

Only a restricted number of large raptors extant in Europe can prey on medium or large-sized birds (e.g. partridge, mallard), even though they can eat smaller vertebrates, such as rodents^46,47^. Diurnal raptors, like the Golden Eagle (*Aquila chrysaetos*) usually pluck their prey and eat it bite by bite, even if the prey is small. Some of these bones are not ingested by diurnal raptors and constitute “uneaten food remains”, while others are ingested and discarded in the form of pellets^36,38,40,41,47–50^. Bones contained in those pellets show higher alterations than those observed in owl pellets, as the articular ends may dissolve completely, often leaving only shafts in the pellets^35,37,41,47,49,50^. On the contrary, owls of the size of the Barn Owl (*Tyto alba*) and the Eagle Owl (*Bubo bubo*) might treat prey in a different manner, depending on its size (when hunting, processing, ingesting or discarding the prey). They swallow the smallest animals entirely, whereas the largest are first separated into portions since large animals cannot be ingested whole^35,48,51^. This differentiation in the treatment of prey can also result in significant variations in terms of alterations of the bones. These differences should be taken into account when trying to determine possible inputs in archaeological assemblages.

For several reasons, the Eagle Owl is a good candidate for exploring this subject. It is a well- known top predator that is extensively distributed throughout Eurasia and can be found in different climatic areas, biotopes and altitudinal ranges. Because of its ubiquitousness, it is a wide-ranging predator whose diet may fluctuate depending on the local environment, the nesting period or the season of the year^48^. It can prey on animals ranging from leporids and other mammals the size of foxes to birds of different dimensions—including raptors—as well as small rodents^48,52–54^. Thus, the possibility of studying the broad-spectrum prey hunted by this pursuer and the different alterations observed on prey bones is of potential interest.

In addition to that, other predators also commonly use the spaces occupied by the Eagle Owl. This owl roosts in caves, shelters or fissures. Some of these spaces could have also been occupied by humans. Consequently, accumulations generated by owls may be mixed up with those generated by humans. Deciphering the Eagle Owl’s taphonomic signature in detail can therefore allow discrimination of its possible accumulations from those with another origin.

The available literature indicates that some authors have previously documented damage on bird bones found in Eagle Owl accumulations. For example, Bocheński and colleagues^35^ compared pellet assemblages of this predator with those originated by the Tawny Owl (*Strix aluco*) and concluded that Eagle Owl accumulations rarely preserve cranial remains, while humerus and tarsometatarsus remains were well rendered. Their work described bone damage due to fragmentation, and they concluded that Eagle Owl tends to break the bones of its victims, probably in relation to prey size. They also noted differences in preservation of the proximal and distal parts of the tibiotarsi, noticing the prevalence of distal portions. A few years later, one of the authors of the present study compared fragmentation as well as other damage observed on pigeon remains recovered from Eagle Owl pellets and Peregrine Falcon (*Falco peregrinus*) food remains^40^. A high preservation was noted for the distal tibiotarsi, as well as for the tarsometatarsi and carpometacarpi, which were the elements that presented the least degree of fragmentation. The study also highlighted the presence of digestion traces on most of the breakage surfaces. It also observed an important number of holes related to beak impacts and mainly located on the proximal humeri. Later, De Cupere and colleagues carried out an analysis of Eagle Owl pellets from the Roman Salagassos site, focusing on the examination of Chukar Partridge (*Alectoris chukar*) remains. They pointed out a high survival rate of the tarsometatarsi, humeri and ulnae, as well as high preservation of complete carpometacarpi. As other authors stated, they documented a heavy underrepresentation of proximal tibiotarsi and recurrent breakage of the long bones^51^. They registered traces of digestive damage on the articular ends of long bones, as well as rounded or thinned edges on the shafts. Nevertheless, they did not present absolute values for the damage representation and, nor did they describe other possible modifications on the bones. These last two studies focused their descriptions on medium-sized birds, and little is known about smaller birds consumed by this predator.

Still later, Alonso and colleagues attempted to describe an Eagle Owl accumulation located near the Spanish Pyrenees from a taphonomic perspective^6^. As in the previous studies, they noted a moderate fragmentation of the bone assemblage. They also pointed out the completeness of the tarsometatarsi and carpometacarpi. A few mechanical modifications were recorded, mainly associated with the sporadic intrusion of a small mammalian carnivore in the cave. The accumulation seems to be a mixture of ingested and non-ingested Eagle Owl remains. Nevertheless, the description exposed the general trends of representation, as it focused on the spatial distribution of the bone modifications. No distinction was made among different bird species.

In light of these reports, the present work proposes a new perspective based on prey size when facing the analysis of bird bone accumulations. When increasing size, animals are prone to suffer more modifications such as fragmentation when consumed by predators. This hypothesis is tested in the present research. To implement this viewpoint, a modern Eagle Owl nest collection recovered at Saint-Vincent-la-Commanderie (Auvergne-Rhône-Alpes region, south-eastern France) has been revised and completed. Part of the bones included in this work were ones previously published in the above-mentioned work^1,40^. Now, birds other than pigeons belonging to the same assemblage are also included to explore, in greater depth the role of prey size in bone modifications.

## Results

### Global prey traits

**Figure 1:**
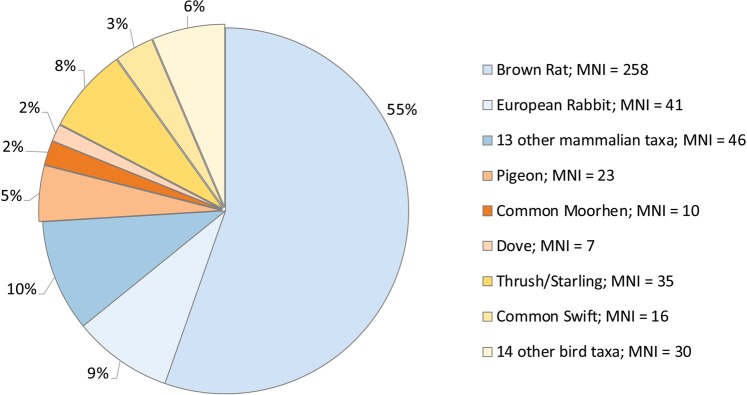
The taxon spectrum from the pellets recovered at Saint-Vincent-la-Commanderie, showing the broad dietary range of the Eagle Owl. Data from mammals according to P. Bayle, pers. comm.

The faunal spectrum from Saint-Vincent-la-Commanderie reflects the generalist predator behaviour of the Eagle Owl. According to P. Bayle (personal communication), mammals are preferred prey over birds. The brown rat (*Rattus norvegicus*) represents more than 50% of the assemblage, followed by the European rabbit (*Oryctolagus cuniculus*) and other mammals mostly of small size. Birds represent nearly 25% of the total recovered individuals (Fig. 1). Among these, a minimum of 19 taxa were identified (Table 1). The thrush/starling genera (*Turdus/Sturnus)* and pigeons (*Columba* sp.) are the most rendered taxa (28.5% and 18.7% of the total MNI, respectively), followed by the Common Swift (*Apus apus*; accounting for 13%), the Common Moorhen (*Gallinula chloropus*; accounting for 8.1%) and doves (*Streptopelia* sp.). From the total 123 individuals identified, 98.4% (MNI = 121) are adults. After their determination, taxa were classified by size categories, so that the most representative species were divided into five groups (see Supplementary Table S1).

**Table 1:** The number of identified specimens (NISP), minimum number of elements (MNE) and minimum number of individuals (MNI) for bird taxa documented at Saint-Vincent-la-Commanderie.

MNI	NISP	MNE	MNI	MNI ad	MNI im
*Anas acuta*	22	15	1	1	—
*Alectoris rufa*	6	5	1	1	—
*Coturnix coturnix*	10	10	1	1	—
*Columba* sp.	325	259	23	22	1
*Streptopelia* sp.	73	66	7	7	—
*Apus apus*	118	107	16	16	—
*Gallinula chloropus*	77	70	10	10	—
*Rallus aquaticus*	10	10	3	3	—
*Vanellus vanellus*	2	2	1	1	—
*Accipiter nisus*	4	3	1	1	—
*Tyto alba*	25	22	4	4	—
*Asio otus*	24	21	5	4	1
Strigiformes undet.	11	11	—	—	—
*Upupa epops*	1	1	1	1	—
*Picus viridis*	7	7	2	2	—
*Falco tinnunculus*	12	12	2	2	—
*Corvus corone*	7	7	1	1	—
*Corvus monedula*	8	8	2	2	—
*Garrulus/Pica*	18	16	3	3	—
*Turdus/Sturnus*	293	260	35	35	—
Passeriformes msz	1	1	1	1	—
Passeriformes ssz	15	15	3	3	—
Aves large size	2	2	—	—	—
Aves medium size	23	21	—	—	—
Aves small size	2	2	—	—	—
TOTAL	1096	953	123	121	2

msz = medium size. ssz = small size. ad = adult; im = immature. Classification according to Clements 2019 version (http://www.birds.cornell.edu/clementschecklist/overview-august-2019/).

### Anatomical representation

In total, 1237 avian specimens were recovered. The present work focuses on the analysis of 1096 specimens corresponding to avian long bones (including coracoids and scapulae). This choice was driven by: (1) the abundance and the determinability of these bones in the archaeological record, and (2) the fact that the authors themselves did not perform the actual collection or processing of the pellets, so the possibility of some biases regarding small fragments, and particularly those from the cranial and axial skeleton, cannot be totally ruled out.

**Figure 2:**
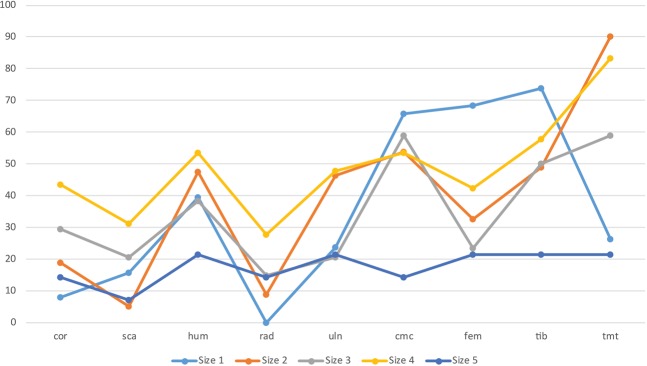
Percentage of relative abundance (%RA) of avian remains recovered at Saint-Vincent-la-Commanderie, classified by size categories. cor: coracoid; sca: scapula; hum: humerus; rad: radius; uln: ulna; cmc: carpometacarpus; fem: femur; tib: tibiotarsus; tmt: tarsometatarsus.

Concerning the relative abundance of elements (Fig. 2), similarities and variations are noticed according to bird size. For each size, the wing-to-leg ratio shows a slight predominance of leg elements (42.9% for size 1; 45.8% for size 2; 47.3% for size 3; 45.1% for size 4; and 30.8% for size 5), but this trend is not statistically significant (see Supplementary Table S2). The medium-sized categories (sizes 2, 3 and 4) present similar trends in skeletal representation. The tarsometatarsus is, by distance, the most frequent element in sizes 2 (90%), 3 (58.8%) and 4 (83.3%). On the contrary, size 1 seems to follow a different trend, with a high representation of tibiotarsi (73.7%) and femora (68.4%); while tarsometatarsi are less abundant (26.3%). All categories present important values of existing elements for carpometacarpi (size 1 = 65.8%; size 2 = 53.6%; size 3 = 58.8%; size 4 = 53.3%), with the exception of size 5 (14.3%). These patterns are clearly conditioned by the most representative species appearing in each size category, which mark the general trends in each group (e.g. the Common Swifts for size 1; thrushes/starlings at size 2; doves for size 3; pigeons and moorhens for size 4). In the case of the largest size category (size 5), the percentages of representation are not strong enough to extract solid conclusions, as the absolute numbers in this group are low (see Supplementary Table S3).

Despite these general trends, within the size 4 some differences can be noticed between the pigeon and the moorhen. Both taxa follow similar trends of representation, but pigeons present higher significant values on the carpometacarpi (76.1% for pigeon; 35% for moorhen), tibiotarsi (73.9% and 50%, respectively) and scapulae (45.7% and 10%, respectively) according to the Z-test (*p*-value < 0.01). Other elements, such as coracoids, femora and tarsometatarsi also differ significantly between both species, with *p-*values < 0.05.

Examination of the proportional representation of long bones reveals no significant preponderance of proximal or distal ends (see Supplementary Table S2). Only the tibiotarsi (for sizes 3 and 4) shows a clear predominance of distal articulations. The proximal to distal ratios for the tibiotarsi are 26.1% (size 3) and 32.9% (size 4), which result in a prevalence of distal specimens. However, the results of a Z-test only show significant differences in the case of size 4 (size 3: *p*-value > 0.05; size 4: *p*-value < 0.05). The other size categories have close to equal values for the proximal and distal ends.

### Fragmentation

**Figure 3:**
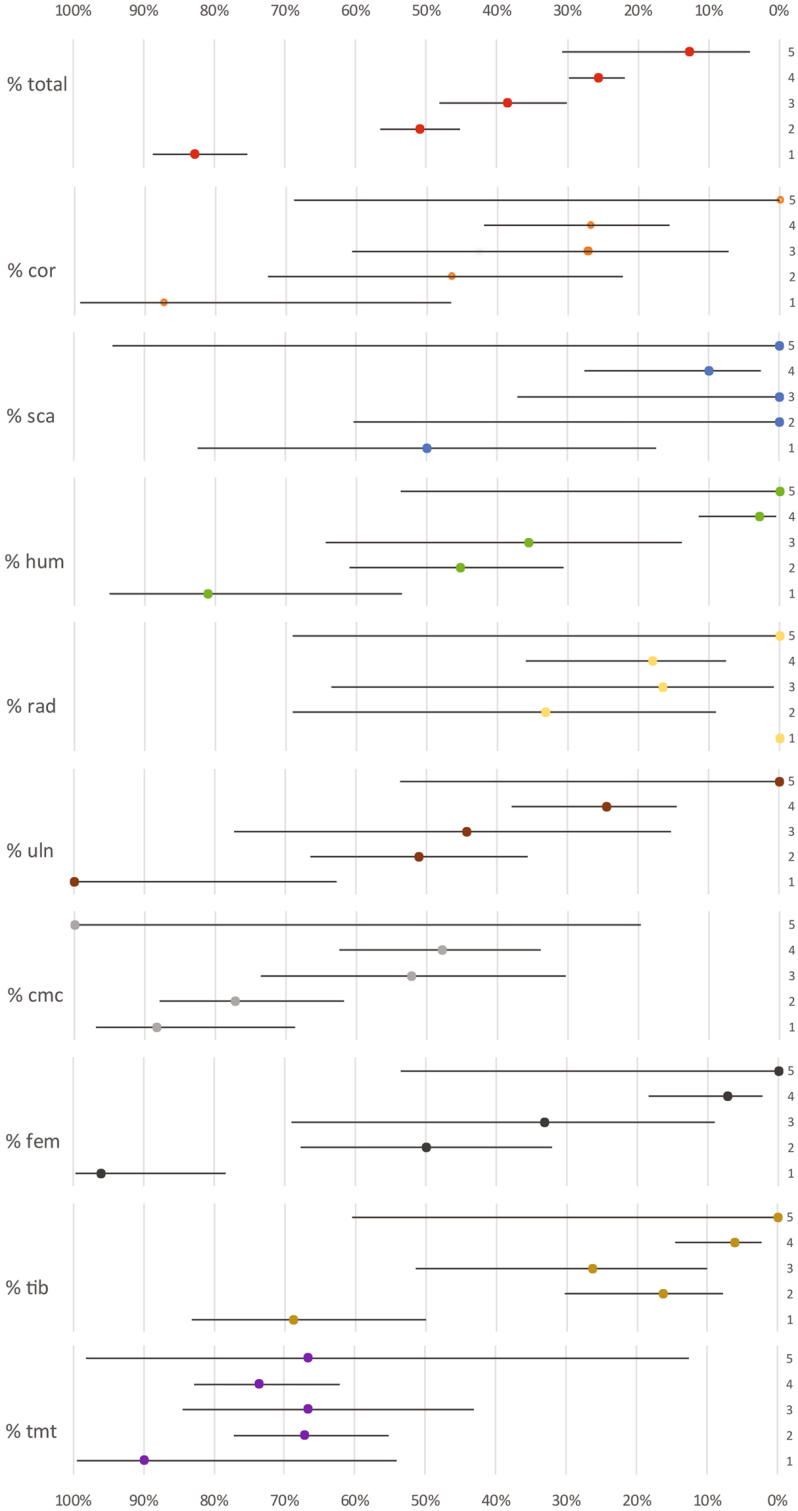
Percentage of bone completeness of different elements and size categories documented at Saint-Vincent-la-Commanderie. Confidence intervals are shown for each item according to the sample. 1–5 refer to bird sizes.

Considering the general percentage of long bone completeness, 41.1% of elements are complete. According to the criteria established by Bocheński^47^, it corresponds to a moderate degree of fragmentation. Substantial differences are highlighted in relation to prey size (Fig. 3). A clear tendency towards fragmentation is evident with the increasing weight of the prey (size 1 = 82.9%, size 2 = 50.9%, size 3 = 38.7%, size 4 = 25.7%, size 5 = 12.9% of the complete elements). A closer look reveals also important variations according to the anatomical element considered. All size together, tarsometatarsi (71%) and carpometacarpi (65.7%) are the most complete, while the scapulae (13.5%), radii (19.6%) and tibiotarsi (21.7%) are highly fragmented. Despite the generally decreasing number of complete elements with increasing size, the tarsometatarsus is an element found complete at high percentages in all size categories, followed by the carpometacarpus (Fig. 3). Notwithstanding, the proportion of complete elements according to prey size is not always systematic. Clear and abrupt variances are observed between the smallest categories (sizes 1–3) and the largest (sizes 4–5), especially regarding the humeri, femora and tibiotarsi. For size 3, the percentages of entire bones for the humerus, femur and tibiotarsus are 35.7%, 33.3% and 26.3%, respectively. Conversely, for size 4, the values of complete elements for those bones decrease to 3%, 7.3% and 6.3%. These differences are statistically significant (*p*-value < 0.05).

Additionally, within the size 4 group some considerations should be noted, since differences among species of similar size are evident (see Supplementary Fig. S1). Moorhen coracoids are recovered complete in high proportions (80%) compared to the pigeon coracoids (12.5%). By contrast, the tarsometatarsi are found complete in greater proportions for pigeons (86.9%) than for moorhens (36.8%).

Among the fragmented remains, 674 fractures are determined as green fractures and accounted for 92.3% of the total fragmented elements. All size categories present values of fresh breakage higher than 93%, with the exception of the smallest size (33.3% for size 1; 93.6% for size 2; 95.2% for size 3; 96.8% for size 4 and 100% for size 5 fractures). The low values for size 1 are explained by the important numbers of modern breakages observed on these bones, probably due to their fragility.

Another important point to note is that some elements are found attached to others by tendons. Attachments are observed in the assemblage at variable percentages (26.6% for size 1; 5.8% for size 2; 19.9% for size 3; 15.4% for size 4; and 48.1% for size 5). The relatively high percentage of attached elements in size 1 deserves particular attention, as 87.5% of the total coracoids in this group are recovered attached to another element (humeri and scapulae). The same condition is observed for the scapulae (75%), humeri (50%) and tibiotarsi (40%) of this size category. Despite the high values present for size 5, the number of total elements represented is low, which makes this proportion unreliable for extracting solid conclusions.

### Surface modifications

From a taphonomic point of view, an important number of specimens displays some type of surface modification. Modified bones represent 83.2% of the specimens and exceed 81% of the total remains for each size category, with the exception of the smallest one (size 1), where they only account for 62.9%. The most common modifications include mechanical modifications, such as pits/punctures, crenulated edges and digestion.

Pits and punctures are present in 7.7% of the total assemblage. Crenulated edges are also observed in a relatively high percentage (4.7%). However, as with fragmentation, the presence of mechanical modifications seems to be directly related to prey size, since the smallest categories only show a few or no beak impacts on bones (Table 2). None of size 1, 2.2% of size 2, 2.5% of size 3, 14.3% of size 4 and 9.7% of size 5 specimens show pits and punctures. If considered together with crenulated edges, the values still follow similar trends (size 1: 0%, size 2: 2.8%, size 3: 5%, size 4: 18.8%, size 5: 9.7%).

**Table 2:** Bone surface modifications, including the total modified remains, the number and (percentage) of pits/punctures, crenulated edges and digestive damage.

**SIZE 1**	**cor**	**sca**	**hum**	**rad**	**uln**	**cmc**	**fem**	**tib**	**tmt**	**Total**
NR	8	8	16	0	9	26	26	32	10	135
Pits/punctures (%)	0 (−)	0 (−)	0 (−)	0 (−)	0 (−)	0 (−)	0 (−)	0 (−)	0 (−)	0 (−)
Cr edge (%)	0 (−)	0 (−)	0 (−)	0 (−)	0 (−)	0 (−)	0 (−)	0 (−)	0 (−)	0 (−)
Digestions (%)	5 (62.5)	7 (87.5)	9 (56.3)	0 (−)	6 (66.7)	21 (80.8)	13 (50)	20 (62.5)	4 (40)	85 (62.9)
**SIZE 2**	**cor**	**sca**	**hum**	**rad**	**uln**	**cmc**	**fem**	**tib**	**tmt**	**Total**
NR	15	4	44	9	43	44	32	49	76	316
Pits/punctures (%)	0 (−)	0 (−)	1 (2.3)	0 (−)	3 (7)	0 (−)	1 (3.1)	0 (-)	2 (2.6)	7 (2.2)
Cr edge (%)	0 (−)	0 (−)	2 (4.5)	0 (−)	1 (2.3)	0 (−)	0 (−)	0 (−)	0 (−)	3 (0.9)
Digestions (%)	13 (86.7)	4 (100)	32 (72.7)	5 (55.6)	35 (81.4)	35 (79.5)	24 (75)	45 (91.8)	60 (78.9)	253 (80.1)
**SIZE 3**	**cor**	**sca**	**hum**	**rad**	**uln**	**cmc**	**fem**	**tib**	**tmt**	**Total**
NR	11	9	14	6	9	21	9	19	21	119
Pits/punctures (%)	0 (−)	0 (−)	2 (14.3)	0 (−)	0 (−)	0 (−)	0 (−)	1 (5.3)	0 (−)	3 (2.5)
Cr edge (%)	0 (−)	0 (−)	3 (21.4)	0 (−)	0 (−)	0 (−)	1 (11.1)	0 (−)	0 (−)	4 (3.4)
Digestions (%)	9 (81.8)	9 (100)	13 (92.9)	5 (83.3)	8 (88.9)	18 (85.7)	9 (100)	17 (89.5)	15 (71.4)	103 (86.6)
**SIZE 4**	**cor**	**sca**	**hum**	**rad**	**uln**	**cmc**	**fem**	**tib**	**tmt**	**Total**
NR	48	30	66	33	57	50	55	80	76	495
Pits/punctures (%)	8 (16.7)	0 (−)	30 (45.5)	0 (−)	6 (10.5)	5 (10)	1 (1.8)	15 (18.8)	6 (7.9)	71 (14.3)
Cr edge (%)	5 (10.4)	2 (6.7)	18 (27.3)	0 (−)	2 (3.5)	4 (8)	3 (5.5)	8 (10)	1 (1.3)	43 (8.7)
Digestions (%)	42 (87.5)	25 (83.3)	61 (92.4)	28 (84.8)	52 (91.2)	45 (90)	52 (94.5)	78 (97.5)	52 (68.4)	435 (87.8)
**SIZE 5**	**cor**	**sca**	**hum**	**rad**	**uln**	**cmc**	**fem**	**tib**	**tmt**	**Total**
NR	3	1	5	3	5	2	5	4	3	31
Pits/punctures (%)	1 (33.3)	0 (−)	1 (20)	0 (−)	0 (−)	0 (−)	1 (20)	0 (−)	0 (−)	3 (9.7)
Cr edge (%)	0 (−)	0 (−)	1 (20)	0 (−)	0 (−)	0 (−)	1 (20)	0 (−)	0 (−)	2 (6.5)
Digestions (%)	3 (100)	1 (100)	5 (100)	3 (100)	5 (100)	2 (100)	4 (80)	4 (100)	3 (100)	30 (96.8)

The results are itemised by the skeletal element and size category.

**Figure 4:**
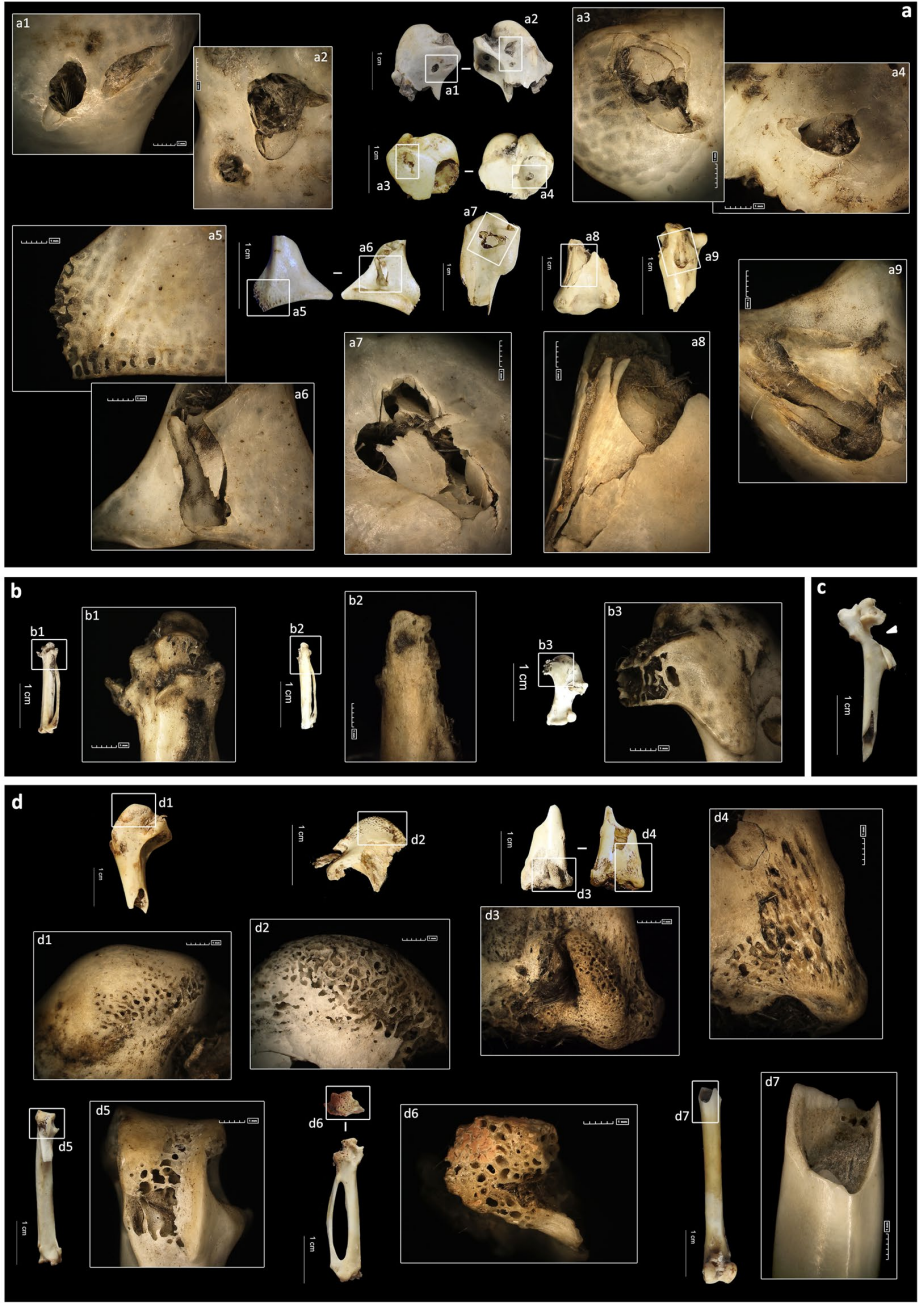
Modifications observed on bones from different sized prey at Saint-Vincent-la-Commanderie, including pits and punctures (**a**), crenulated edges (a8, c, d4) and digestive damage (**b**,**d**). a1/a2 and a3/a4: proximal humeri of *Columba* sp.; a5/a6: coracoid of *Columba* sp.; a7: proximal humerus of *Asio otus*; a8: distal humerus of an undetermined Strigiformes, a9: proximal ulna of *Tyto alba*; (**b**) carpometacarpi (b1, b2) and humerus (b3) of *Apus apus*; (**c**) carpometacarpus of *Columba* sp.; (**d**) humeri (d1, d2, d3/d4), carpometacarpi (d5, d6) and tibiotarsus (d7) of *Columba* sp.

The coracoids, humeri and tibiotarsi are the most modified elements in relation to pits and punctures (Fig. 4), particularly in the case of size 4 (Table 2). The humerus is the bone that presents the highest values when comparing smaller sizes with the biggest ones (in size 3, 14.3% bones have pits or punctures, while in size 4 the proportion increases to 45.5%), and it shows significant differences in relation to prey size (*p*-value < 0.01). Nevertheless, an important point is that within the size 4 group, significant differences exist between the two main taxa component of this group. Most of the perforations counted for this category belong to pigeons (n = 55 from the 78 total perforated specimens). Despite the notable number of remains reported for moorhen, the proportion of perforated bones for that taxon is lower (only four holed remains). This evidence is even more noticeable among humerus remains (54.3% perforated humeri for pigeons and 20% for moorhens), and statistically significant (Z = −1.97, *p*-value < 0.05).

**Figure 5:**
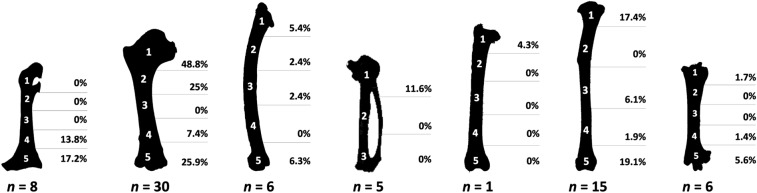
Distribution and percentage of pits and punctures on bones of size 4 category prey by anatomical portions. Only size 4 was considered for the figure, as values were absent or not statistically significant in other categories. Even if other taxa are included in the size 4 group, pigeon bones were used to illustrate bone figures, as they are the most representative in the assemblage. 1: proximal end; 2: proximal shaft; 3: mid-shaft; 4: distal shaft; 5: distal end (for coracoids, humerus, ulna, femur, tibiotarsus and tarsometatarsus). For the carpometacarpus, a simplified division of the proximal end (1), shaft (2) and distal end (3) is used. The percentages indicate the proportion of each bone segment showing such damage in relation to the total portions present in the assemblage. “*n*” indicates the number of bones showing pits and/or punctures.

Pits and punctures are mainly localised at the proximal or distal ends in all elements and categories. As with perforated elements, size 4 seems to be representative in terms of absolute numbers (see Supplementary Table S4). The humerus is specially damaged showing perforations at approximately half of the proximal ends (Fig. 5). On this bone, one quarter of the distal ends and the proximal shaft portion also show such damage. Modifications are multiple or isolated, unilateral or bilateral, depending on the bone. The humeri commonly show multiple and bilateral perforations. Single marks are more common on other long bones.

**Figure 6:**
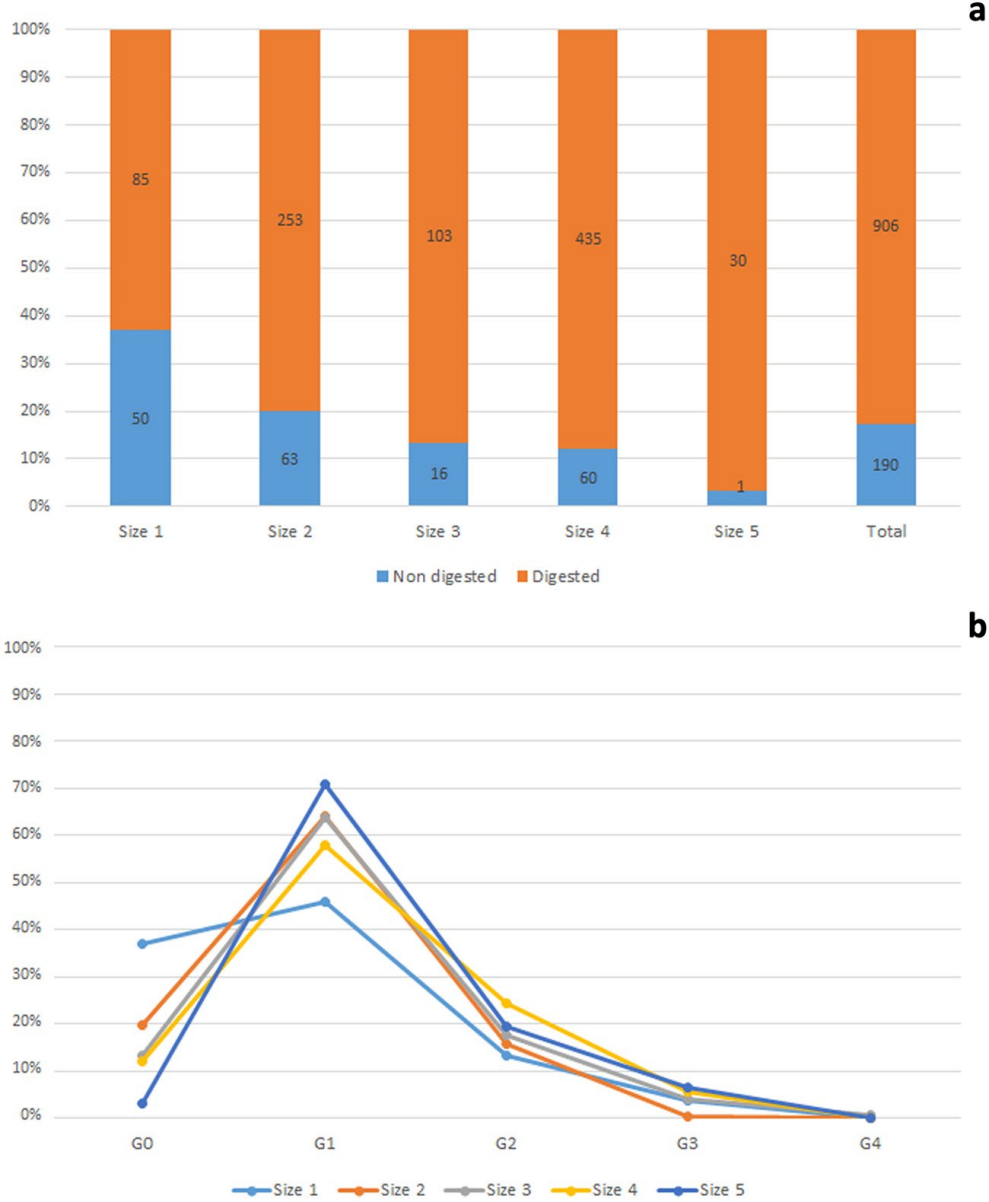
Percentage of digested and non-digested bones recorded at Saint-Vincent-la-Commanderie by size categories (**a**) and their distribution according to the different degrees of digestive damage stated by Andrews^75^ (**b**). Bar numbers refer to the number of remains registered in each case.

Digestive corrosion affects 82.7% of the total remains. All size categories show high proportions of digestive alterations (>80% of digested bones), with size 1 being the exception with only 62.9% of digested bones (Table 2, Fig. 6). Only considering effective of elements higher than 10, the tarsometatarsus is the least altered element (size 1: 40%; size 2: 78.9%; size 3: 71.4%; size 4: 68.4%). Additionally, different degrees of digestion are diagnosed (see Supplementary Fig. S2), with light digestion predominating in all categories (Fig. 6), at 45.9%, 63.9%, 63.9%, 57.8% and 70.9% of the total digested elements from sizes 1 to 5, respectively. Size 3 is the only size presenting a clear extreme degree of corrosion on a humerus fragment. Nevertheless, all the digested fracture edges might not have corresponded to real bone breakage, as extreme corrosion and subsequent articular loss could produce similar patterns at the fracture edges. Therefore, distinguishing extreme epiphysis corrosion from strong corrosion of the fracture edge after the epiphyses was broken is difficult and not always possible.

Digestion is mainly diagnosed at fracture edges (see Supplementary Table S5 for further information), where damage appears as rounded and soft edges. At the articular ends, digestive corrosion is less extended. Both the proximal and distal ends show indistinct dissolution due to the action of gastric acids. Only in the case of the tarsometatarsus is a clear prevalence of digestion evident on the proximal ends, rather than the distal ones, in all groups (Supplementary Table S5). Comparison of species of the same size reveals significant differences for the digestive corrosion between pigeons and moorhens on the carpometacarpus (100% and 42.9% digested, respectively) and the ulna (94.3% and 66.7%, respectively).

## Discussion

The discrimination of predator activities in archaeological accumulations is a crucial issue for understanding the site formation processes and bird–human interactions. Despite the increasing knowledge, much work remains; therefore, many researchers have endeavoured to decipher patterns to characterise the signature of non-human predators on bone assemblages. However, the present data point to the importance of analysing referential assemblages in detail, as many different points are noticed. Simply taking general trends into account may hide other important information that could help with the characterisation of an accumulation, especially in terms of prey size. This variability should be considered when analysing bones, as the damage caused in one category of prey will differ from that observed in another, and this can lead to erroneous diagnoses of an archaeological accumulation.

Some of the results presented here are, by implication, expected. However, the aim of the present work was to describe and systematise an accumulation originated by one of the most common nocturnal raptors taking part in the archaeological record by providing specific values that would be useful for future approaches. Previous work already foresaw that Eagle Owl ethology would influence the modifications observed on prey bones, as differential treatment of prey according to their size is noted^35,55^. The present analysis confirms the previous studies that relied on the importance of prey size when facing archaeological assemblages and shows clear differences when tracking bone damage on different prey^1,43–45^. The fact that an important number of remains are affected by modifications other than fragmentation is not surprising, as the specimens came from pellets. Nevertheless, the main differences are noticed regarding fragmentation and mechanical modifications. While some animals will be eaten whole, as their size allows it, others will undergo a different consumption path because: (1) they cannot be ingested whole, and (2) other nutritional values could be considered in larger prey. This consumption process necessarily influences the state of bone preservation.

The previous studies carried out in bird bone modifications generated by the Eagle Owl focused on fragmentation^35^, although a few authors attempted to describe some other taphonomic traits^6,40,51^. They did not focus on the influence of prey size on the modification pattern^6,51^. The present research shows that the described and documented patterns meet the above-mentioned criteria, as global bone breakage and mechanical modifications increase in relation to prey size due to the need to separate prey into smaller pieces for ingestion. However, when anatomical elements are considered individually, note that the number of specimens of the largest size is low and not sufficient to reach statistical significance (see Fig. 3). Regardless, the percentage of bones with some kind of modification is remarkable and suggests trends in representation that would be expected if the sample was larger.

Although the hypothesis raised seems to meet expectations, some differences remain unexplained. This is particularly the case for the smallest category (size 1), which normally shows results that clearly stand apart from the rest. In the case of anatomical representation, size 1 is distinguished by the extremely low values for tarsometatarsi and the abundance of femora and tibiotarsi. These values deviate from the general mean of representation and from the results previously published by different authors^6,35,51^. One point that should be taken into account is that, in our sample, size 1 is almost entirely represented by the Common Swift, whose tarsometatarsus is very small. This determination might be even more difficult in the case of the smallest bones. The classification of skeletal elements by size minimises the biases when classifying taxa in general categories, but researchers must also consider the impossibility of identifying some small broken elements at the anatomical level. Therefore, some small-sized bones may possibly pass unnoticed when trying to sort them.

Comparison of the present results with previously published data on Eagle Owl accumulations revealed similar patterns in terms of fragmentation^6,35^. However, this is only true when considering the general trends. A closer look at those categories from both extreme points of the sample (sizes 1, 4 or 5) shows that the values deviated from the mean. Bone breakage is moderate in size 1, while it drastically increases from size 4 and the above, particularly referring in the case of some of the long bones (humerus, femur and tibiotarsus). This behaviour is commonly observed in Eagle Owl prey specimens recovered from nest sites, as female owls tend to dismember larger prey and give small pieces to their chicks^35^. These bones bear an important quantity of meat but, because of their dimensions, they have to be broken for ingestion. The carpometacarpus and tarsometatarsus are also considered long bones, but their degree of fragmentation is lower in all categories, probably because of their small quantity (or lack) of meat. The lowest digestion observed on the tarsometatarsus could also be due to the scales covering it. Consequently, these bones are not nutritionally attractive, so they are less subject to damage. De Cupere and colleagues also appreciated these distinctions in the Chukar Partridge analysis they performed^51^ and their results can be compared to and are consistent with those obtained for the size 4 category in the present study.

De Cupere and colleagues, as well as Bocheński and his partners, also recorded the importance of distal tibiotarsi in the case of medium-sized prey birds^35,51^. They explain the prevalence of distal ends because of the stoutness of some articular parts. This representation is probably not related to Eagle Owl ethology, as the importance of the distal tibiotarsus has been recorded in other predator assemblages, such as in the uneaten remains from the Imperial Eagle or the Gyrfalcon^36,38,47^. Therefore, other explanations, such as differential survivorship, might be valid. Dirrigl previously noted that the bone mineral density volume is considerably lower in the proximal end than in the distal end of the tibiotarsus in Wild Turkey (*Meleagris gallopavo*)^56^. Although this bird is substantially larger than partridges or pigeons, they all have a similar morphology, which makes comparison possible.

Size categories based on weight have been recognised as a crucial factor when determining bone damage in the case of the Eagle Owl, but other factors may also affect it. One potential influence are bone morphometric factors. As observed, pigeons and Common Moorhens present divergent degrees of fragmentation on some bones, such as the coracoids and tarsometatarsi. On the one hand, the moorhen coracoid is noticeably smaller and more robust, which would explain its predisposition to less breakage. On the other hand, the tarsometatarsus is larger in the moorhen than in the pigeon, which favours breakage. Furthermore, even though the degree of humerus fragmentation is similar in both species, pigeon humeri are much more altered by mechanical modifications (pits and punctures) than are those of moorhens. In this sense, some substantial differences can be appreciated. For instance, the pigeon humerus has a particular morphology. On its proximal part, it carries a very blunt head, higher than the tubercle, on the proximal extremity. The pneumatic fossa is large and rounded. Overall, this results in a wider morphology of the proximal end. Its shaft is short and has an extensive development of the medullary cavity that continues with a more or less constant width until the distal end^57^. Conversely, the Common Moorhen humerus is slim and less blunted on its proximal end, as the crista pectoralis does not extend dorso-cranially. The shaft is proportionally longer and thinner. Consequently, when comparing both taxa, the squat morphology of pigeon humeri makes them more prone to suffer damage since the contact surface is larger. Thus, the observation that most of the mechanical modifications documented on bones were located on pigeon humeri is not unexpected, as was previously noted^40^. In addition, as pigeons are the most frequent taxon in the size 4 group, the abundant values of mechanical modifications for this category would be conditioned by their predominance, displaying values of even greater importance than in the case of large-sized birds.

The low values of digestive corrosion observed in the smallest category are another point of discussion. Since small-sized birds can be ingested whole, they might be expected to show high percentages of alteration. However, this complete ingestion could result in protection of the bones by the meat, the skin and feathers covering them, thereby reducing damage and the percentage of alteration. The preservation of attached elements in some categories corroborates this hypothesis, because tendons can protect bone ends from severe alterations. On the contrary, the carpometacarpi of size 1 suffered important damage when compared with other bones. The absence of meat in this area of the wing may expose the bone to more damage after ingestion, even though skin could protect it.

The proximal ends of some bones from the size 4 category also show higher percentages of digestive corrosion when compared with the previously published data on pigeon bones^40^. This could be a result of inter-observer appreciation of the very first stage of digestion. The tendons that cover part of these articular ends also hinder first observations of them. For the present work, the tendons were pulled off to observe in detail these partially covered areas.

The current analysis, and as also perceived by De Cupere^51^, revealed that digestive dissolutions first affect the fracture edges and later spread to the proximal and distal ends as the degree of damage increases. On complete elements, the first sign of digestion is observed on the epiphyses, as identifying digestive damage on long-bone shafts is difficult if they are not broken before intake. The low percentage of fresh breakage observed in size 1 considerably reduces the number of fracture edges likely to experience damage, so digestive corrosion may pass unnoticed in some cases. In addition, although digestive corrosion is important in the accumulation, the degree of digestion is light and does not always affect bone epiphyses, which further complicates its diagnosis.

Despite the light character of digestive corrosion on the assemblage, an accurate diagnosis of digestive degrees may sometimes be controversial. Some bones present fracture edges with a strong degree of digestion and show an absence of the bone epiphysis (Supplementary Fig. S2 - c6 and d11). This presentation complicates assessment of whether the epiphyses disappeared because of extreme digestion or whether the bones were broken before being ingested, causing damage at the fracture edges. If the first situation is considered, the bones should show evidence of an extreme degree of corrosion, thereby increasing the previously calculated percentage. In the same way, the analysis should take into account that relating a modification to a specific activity is easier in modern collections whose origin is known. On the contrary, the remains found in archaeological assemblages undergo other processes that might hinder the observation of digestion on certain parts of the bone or that can be confused with other post-depositional processes (i.e. erosion)^58,59^.

Many factors can influence bone damage and its diagnosis in bones from an assemblage. The predator itself and its ethology influence the treatment of prey. The results of the present work conclude how prey size affects some of the damages caused by the Eagle Owl. Important differences can be appreciated in terms of fragmentation and surface damage on bones depending on the prey dimensions. Nevertheless, other factors, such as bone morphometry, also affect the degree of alteration. For this reason, understanding the particularities of different taxa represented in an assemblage becomes crucial. In that sense, actualistic research may help to provide a better understanding of the behaviour of many predators and to improve the characterisation of their taphonomic signatures.

By contrast, all these specificities reveal the complexity of pointing out precise characters for predator determination. Even taking into account all these variables, actualistic studies are based on an idyllic situation, where bone loss is very low and post-depositional processes do not have time to alter significantly bones. The archaeological reality shows the difficulty of finding similar situations in ancient assemblages^60^. Archaeological remains suffer the degradation of time and are subject to different post-depositional processes that alter bone surfaces and hinder their preservation. Hence, not all the modifications documented in archaeological accumulations can be diagnosed with total certainty, such as in the case of Saint-Vincent-la-Commanderie. As proposed by other scholars, the development of multi-taxon approaches^43,61^ and inter-observer^62^ analyses could provide further methods to surmount this barrier.

## Methods

The bird remains analysed for the present work come from pellets recovered directly from the vicinity of an Eagle Owl nest by Patrick Bayle in the Drôme area (South-Eastern France). The pellets were disaggregated and a preliminary determination of specimens was carried out before the present analyses.

The analysed remains were anatomically and taxonomically identified using both bibliographic references^63–66^ and actual bird collections held at PACEA – Université de Bordeaux laboratory and the Muséum National d’Histoire Naturelle of Paris. The number of identified specimens (NISP) and the minimum number of elements (MNE) were used to estimate a minimum number of individuals (MNI)^67^ and the percentage of relative abundance in the assemblage (%RA)^68^. Immature and adult individuals were distinguished using the degree of ossification of cortical tissues^69,70^. After the initial identification, the remains were classified by taking into account five larger categories based on the weight of prey (Supplementary Table S1): size 1 (<50 g); size 2 (51–150 g); size 3 (151–250 g); size 4 (251–500 g); size 5 (>500 g). The wing-to-leg^71^ ratio was computed to assess possible differences regarding anatomical representation by size. It is the percentage resulting from the division of the total wing remains (humeri, ulnae and carpometacarpi) by the sum of the wing and leg remains (femora, tibiotarsi and tarsometatarsi). The presence of proximal fragments in relation to distal ends was computed following the Bocheński criteria^47^, dividing the total proximal parts (whole bones + proximal ends) by the sum of proximal and distal parts (whole bones + distal ends), and expressed as a percentage. The degree of fragmentation was also considered, following standards previously stated, to distinguish between low (<30% of complete bones), moderate (30–60%) and high (>60%) degrees of bone completeness^47^.

A Euromex stereomicroscope (Nexius Zoom NZ 1902-P) with magnification up to 45x and a HIROX KH-8700 3D digital microscope with magnification up to 35x were used for the taphonomic analysis of remains. Green fractures were distinguished from dry and modern fractures by the presence of oblique angles and smooth edges^72,73^. Other mechanical modifications, such as beak marks (including pits/punctures, scores, crenulated edges and longitudinal cracks)^40,50,74^, were recorded, taking into consideration their location along the bone (proximal or distal ends, shafts portions) and their distribution (isolated, unilateral, bilateral). Digestion was assessed considering five corrosion degrees: 0 (null), 1 (light), 2 (moderate), 3 (strong) and 4 (extreme)^75^ (see Supplementary Fig. S2). The location of damage along the bone (proximal/distal end, fracture edge) was also considered.

The bone representation and fragmentation in relation to prey size were assessed by calculating the bilateral confidence interval of a proportion and applying Wilson’s method with continuity correction^76,77^. This interval was computed to evaluate the range of variation of a *p*-value in a sample in order to determine if significant differences could exist among the different values obtained. In addition, the Z-test was applied for those values that might show significant differences between sizes^78^.

## Supplementary information

Supplementary Information

## Data Availability

The datasets generated during and/or analysed during the current study are available from the corresponding author on reasonable request and/or included in this published article (and its Supplementary Information Files).
