# Supplementary material for: Prey size as a critical factor for bird bone taphonomy in Eagle Owl (Bubo bubo) pellets
Source: Sci Rep. 2019 Dec 16;9:19200. doi: 10.1038/s41598-019-55721-7 (PMC6915716; doi:10.1038/s41598-019-55721-7)

Prey size as a critical factor for bird bone taphonomy in Eagle Owl (*Bubo bubo*) pellets - [Supplementary Information]

Anna Rufà[1*]
Véronique Laroulandie[1]

[1] PACEA - UMR 5199 CNRS, Université de Bordeaux, Bâtiment B2. Allée Geoffroy Saint Hilaire CS 50023, 33615 Pessac Cedex, France

*Corresponding author: arufabonache@gmail.com

Supplementary Table S1. Classification of taxa by each category according to their size and average weight.

**Size**	**Weight (g)**	**Scientific name**	**Common name**
Size 1<br>NISP = 135	<50	*Apus apus*<br>Small Passeriformes	Common swift<br>Small perching birds
Size 2<br>NISP = 316	51-150	*Coturnix coturnix*<br>*Rallus aquaticus*<br>*Upupa epops*<br>*Turdus/Sturnus*	Common quail<br>Water rail<br>Eurasian hoope<br>Thrush/Starling
Size 3<br>NISP = 119	151-250	*Accipiter nisus*<br>*Falco tinnunculus*<br>*Vanellus vanellus*<br>*Picus viridis*<br>*Streptopelia* sp.<br>*Garrulus/Pica*<br>Medium Passeriformes	Eurasian sparrowhawk<br>Common krestel<br>Northern lapwing<br>European green woodpecker<br>Dove<br>Jay/Magpie<br>Medium perching birds
Size 4<br>NISP = 495	251-500	*Asio otus*<br>*Tyto alba*<br>Strigiformes undet.<br>*Alectoris rufa*<br>*Columba* sp.<br>*Gallinula chloropus*<br>*Corvus monedula*	Long-eared owl<br>Barn owl<br>Owl indet.<br>Red-legged partridge<br>Pigeon<br>Common moorhen<br>Western jackdaw
Size 5<br>NISP = 31	>500	*Anas acuta*<br>*Corvus corone/frugilegus*	Northern pintail<br>Carrion crow/Rook

Supplementary Table S2. Z-test values on wing-to-leg and proximal-to-distal fragments ratios. The real values obtained are provided in the first column (*n*), followed by the total sum of wing and leg or prox(imal)-dist(al) remains. The third and the forth columns refer to the expected number of remains, in order to calculate the deviation from the expected percentage (50%). The fifth column (Z-value) refers to the Z-values obtained. The *p*-values (last column) do not reveal any statistically significant differences.

**wing-to-leg**	***n* wings**	**Total wings+legs**	**Expected wings**	**Total expected wings+legs**	**Z-value**	***p*-value**
Size 1	51	119	60	120	-1,107	$0.26<p<0.27$
Size 2	131	288	144	288	-1,084	$0.27<p<0.28$
Size 3	44	93	47	94	-0,368	$0.71<p<0.72$
Size 4	173	384	192	384	-1,373	$0.16<p<0.17$
Size 5	4	13	7	14	non-calculable	non-calculable
**proximal-to-distal**	***n* proximal**	**Total prox+dist**	**Expected proximal**	**Total expected prox+dist**	**Z-value**	***p*-value**
Size 1	127	247	124	248	0,315	$0.75<p<0.76$
Size 2	253	474	237	474	1,040	$0.29<p<0.30$
Size 3	84	164	82	164	0,221	$0.82<p<0.83$
Size 4	305	597	299	598	0,376	$0.70<p<0.71$
Size 5	18	33	17	34	0,372	$0.70<p<0.71$

Supplementary Table S3. Minimum Number of Elements and percentage or Relative Abundance (in parentheses) by size categories and in the specific case of the pigeon and the moorhen.

**%RA size**	**cor**	**sca**	**hum**	**rad**	**uln**	**cmc**	**fem**	**tib**	**tmt**
Size 1	3 (7.9)	6 (15.8)	15 (39.5)	0 (0)	9 (23.7)	25 (65.8)	26 (68.4)	28 (73.7)	10 (26.3)
Size 2	15 (18.8)	4 (5)	38 (47.5)	7 (8.8)	37 (46.3)	43 (53.8)	26 (32.5)	39 (48.6)	72 (90)
Size 3	10 (29.4)	7 (20.6)	13 (38.2)	5 (14.7)	7 (29.6)	20 (58.8)	8 (23.6)	17 (50)	20 (58.8)
Size 4	39 (43.3)	28 (31.1)	48 (53.3)	25 (27.8)	43 (47.8)	48 (53.3)	38 (42.2)	52 (57.8)	75 (83.3)
Size 5	2 (14.3)	1 (7.1)	3 (21.4)	2 (14.3)	3 (21.4)	2 (14.3)	3 (21.3)	3 (21.4)	3 (13)
Pigeon	24 (52.2)	21 (45.7)	32 (69.6)	15 (32.6)	27 (58.7)	35 (76.1)	26 (56.5)	34 (73.9)	45 (97.8)
Moorhen	5 (25)	2 (10)	9 (45)	4 (20)	9 (45)	7 (35)	5 (25)	10 (50)	19 (75)

Supplementary Table S4. Total number of portions counted by each long bone from size 4 category and the number of perforated portions for each bone (values in parentheses). The last column refers to the total perforated remains in relation to the total bones present in this group. For the carpometacarpus and the scapula, a simplified division of portions is used (1-3) due to their morphology.

	**Portion 1**	**Portion 2**	**Portion 3**	**Portion 4**	**Portion 5**	**Total**
coracoid	32 (-)	33 (-)	18 (-)	29 (4)	29 (5)	48 (8)
scapula	27 (-)	23 (-)	8 (-)	-	-	30 (-)
humerus	41 (20)	20 (5)	19 (-)	27 (2)	27 (7)	66 (30)
radius	23 (-)	23 (-)	28 (-)	14 (-)	14 (-)	33 (-)
ulna	37 (2)	42 (1)	41 (1)	32 (-)	32 (2)	57 (6)
carpometacarpus	43 (5)	48 (-)	35 (-)	-	-	50 (5)
femur	23 (1)	21 (-)	16 (-)	31 (-)	35 (-)	55 (1)
tibiotarsus	23 (4)	29 (-)	49 (3)	53 (1)	47 (9)	80 (15)
tarsometatarsus	59 (1)	64 (-)	70 (-)	72 (1)	72 (4)	76 (6)

Supplementary Table S5. Distribution of digestive damage between the proximal end (dig px), the distal end (dig ds) and fracture edges (dig f). The total proximal ends (px), distal ends (ds) and fracture edges (f) of the assemblage are provided for each bone, as well as the percentage rendered for each element and category.

		px	dig px	% dig px	ds	dig ds	%dig ds	f	dig f	% dig fract
Size 1	cor	8	4	50.0	7	0	0.0	1	1	100.0
	sca	8	1	12.5	-	-	-	7	7	100.0
	hum	15	8	53.3	14	1	7.1	3	0	0.0
	rad	0	0	0.0	0	0	0.0	0	0	0.0
	uln	9	4	44.4	9	4	44.4	1	0	0.0
	cmc	25	17	68.0	24	15	62.5	13	7	53.8
	fem	26	7	26.9	25	10	40.0	2	1	50.0
	tib	27	9	33.3	27	12	44.4	13	8	61.5
	tmt	9	4	44.4	10	1	10.0	2	0	0.0
Size 2	cor	15	7	46.7	7	3	42.9	11	9	81.8
	sca	4	1	25.0	-	-	-	4	4	100.0
	hum	34	17	50.0	29	13	44.8	24	21	87.5
	rad	7	1	0.0	5	2	0.0	6	3	0.0
	uln	35	16	45.7	30	16	53.3	21	19	90.5
	cmc	44	21	47.7	34	9	26.5	25	25	100.0
	fem	25	5	20.0	23	15	65.2	17	13	76.5
	tib	25	20	80.0	30	18	60.0	41	37	90.2
	tmt	64	40	62.5	63	24	38.1	25	20	80.0
Size 3	cor	8	5	62.5	6	5	83.3	8	7	87.5
	sca	9	3	33.3	-	-	-	9	8	88.9
	hum	12	6	50.0	7	5	71.4	11	8	72.7
	rad	5	2	0.0	2	1	0.0	5	5	0.0
	uln	7	5	71.4	6	3	50.0	6	5	83.3
	cmc	16	10	62.5	16	6	37.5	16	15	93.8
	fem	6	6	100.0	6	6	100.0	6	6	100.0
	tib	6	6	100.0	17	12	70.6	15	11	73.3
	tmt	15	10	66.7	20	6	30.0	8	5	62.5
Size 4	cor	32	18	56.3	29	10	34.5	40	36	90.0
	sca	27	14	51.9	-	-	-	27	24	88.9
	hum	41	20	48.8	27	11	40.7	64	58	90.6
	rad	23	8	34.8	14	12	85.7	27	20	74.1
	uln	36	29	80.6	31	19	61.3	49	38	77.6
	cmc	41	33	80.5	32	13	40.6	41	32	78.0
	fem	23	17	73.9	35	26	74.3	51	48	94.1
	tib	23	16	69.6	47	42	89.4	75	71	94.7
	tmt	59	34	57.6	72	34	47.2	27	20	74.1
Size 5	cor	1	1	100.0	1	1	100.0	3	3	100.0
	sca	1	1	100.0	-	-	-	1	1	100.0
	hum	1	1	100.0	4	4	100.0	5	5	100.0
	rad	3	2	0.0	0	0	0.0	3	3	0.0
	uln	3	1	33.3	2	1	50.0	5	5	100.0
	cmc	2	2	100.0	1	0	0.0	2	2	100.0
	fem	2	1	50.0	3	2	66.7	5	4	80.0
	tib	2	2	100.0	2	2	100.0	4	4	100.0
	tmt	3	3	100.0	2	2	100.0	1	1	100.0

Supplementary Figure S1. Percentage of completeness, with its confidence interval, of the proportion provided for the long bones of pigeon (blue) and moorhen (orange).

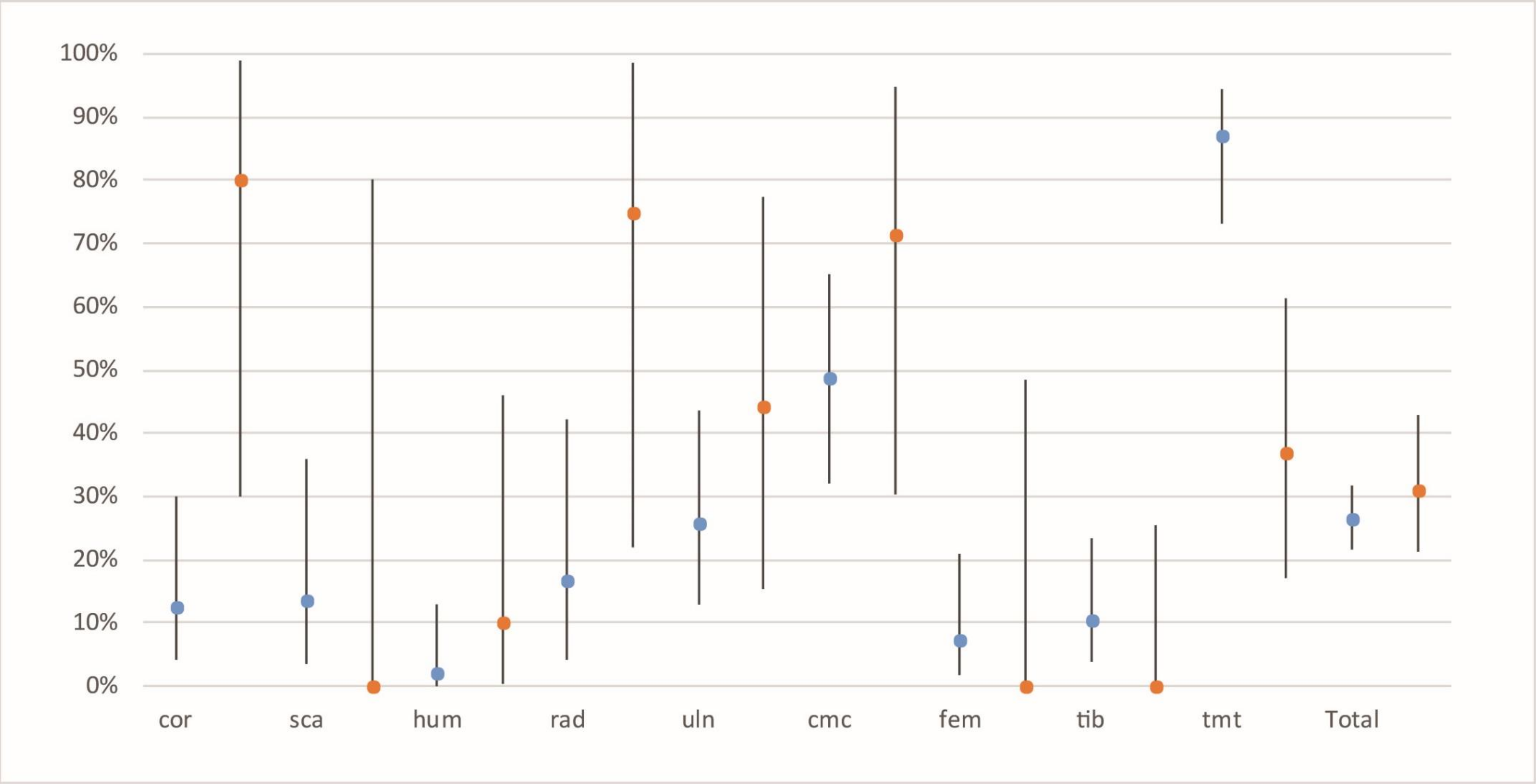

Supplementary Figure S2. Representation of different degrees of digestive damage on long bones of *Columba* sp. documented at Saint-Vincent-la-Commanderie. a) carpometacarpus; b) ulna; c) tibiotarsus; d) tarsometatarsus. Note the continuum of digestion damage from null (c1) to extreme (c6).

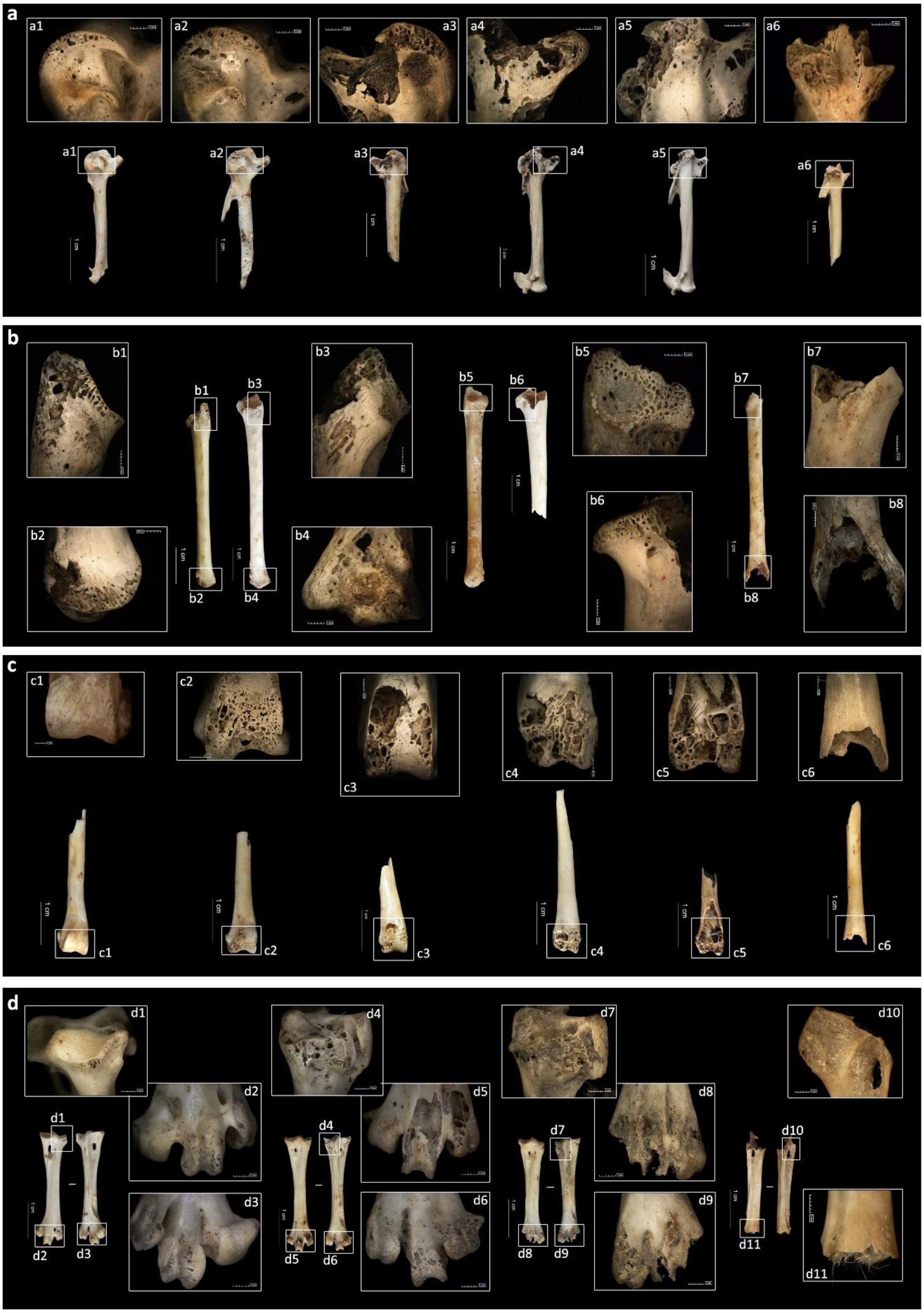

Supplement: Supplementary file 1 — Supplementary Information [file 41598_2019_55721_MOESM1_ESM.pdf]
